# The Reasons behind COVID-19 Vaccination Hesitancy among the Parents of Children Aged between 5 to 11 Years Old in Saudi Arabia

**DOI:** 10.3390/ijerph20021345

**Published:** 2023-01-11

**Authors:** Elham Mohammed Khatrawi, Anwar A. Sayed

**Affiliations:** 1 Department of Medical Microbiology and Immunology, Taibah University, College of Medicine, Madinah 42353, Saudi Arabia; 2 Department of Surgery and Cancer, Imperial College London, London SW7 2AZ, UK

**Keywords:** COVID-19, COVID-19 vaccines, vaccination hesitancy, Saudi Arabia

## Abstract

Simultaneously with the development of the COVID-19 vaccination plan for minors, it is critical to understand the reasons related to parental COVID-19 vaccination hesitancy. This study aims to determine the reasons associated with vaccination hesitancy among parents, and the prevalence and the characteristics of the parents who are hesitant to allow their children aged between 5 to 11 years old to be administered the COVID-19 vaccines. A web-based questionnaire was used to perform this study between May 2022 to September 2022 in Saudi Arabia (SA). Several factors, personal and social, affected the participants’ willingness to vaccinate their children with the COVID-19 vaccines. The age of the parents was found to have a significant impact on their decision to vaccinate their children. Those between the age of 40–49 years of age were the most willing to vaccinate (almost 41%) compared to those 50 years or older who were most resistant to vaccination. Female participants were more resistant to vaccinating their children compared to their male counterparts. Saudis were more resistant to vaccinating their children compared to the non-Saudi participants. Those private sector-employed parents were the most willing to vaccinate (16.6%), followed by those working in the governmental sector (13.8%). About 40.7% of non-healthcare workers were resistant to vaccinating their minor compared to healthcare workers (8.7%). In conclusion, the study presents several factors that affect the parental willingness to vaccinate their children in SA. These factors should be properly addressed when developing public health strategies to promote the COVID-19 vaccination of children in SA.

## 1. Introduction

The pandemic coronavirus disease (COVID-19) has resulted in increasing morbidity and mortality rates, as well as increasing the burden on hospitals and healthcare systems around the world [1]. Precautionary measures were implemented across the globe to limit the transmission of this infection, such as mask-wearing, hand hygiene, social distancing and travel restriction. Numerous countries still encounter waves of COVID-19 cases. In the Kingdom of Saudi Arabia (KSA), the overall number of cases and deaths has exceeded 747,436 and 9008, respectively [2]. Together with the applied safety measures to stop the virus transmission, a COVID-19 vaccine is believed to slow the outbreak, save lives, and minimize the burden on the healthcare system [3,4,5,6,7,8]. Furthermore, COVID-19 control measures were helpful in tackling other infectious conditions, such as tuberculosis [9]. The Ministry of Health (MOH) in KSA launched a vaccination campaign via a mobile phone App called “Sehaty” that helps people to register and get the vaccine. Moreover, many vaccine centres were founded in cities and villages across the country (MOH, no date). The campaign was begun on 17 December 2020, providing Oxford/AstraZeneca and Pfizer-BioNTech COVID-19 immunization and endeavored to immunize all the residents and citizens, free of charge [10].

On 29 October 2021, the Food and Drug Administration (FDA) allowed children aged between 5 to 11 years old to administer the Pfizer-BioNTech vaccine in two doses (10 µg and 0.2 mL for each dose) 21 days apart. This dose is less than the 30 µg administered by adults and older children [11]. Only the Pfizer-BioNTech vaccination is licensed to be given to children between 5 and 17 years old [12,13]. It is essential to vaccinate this age group to control both the transmission of this virus and the severity of this infection among this age group because children are also prone to catch COVID-19 [5,13,14,15,16]. In the United States, there are about 28 million children aged 5 to 11 years old, and approximately 2 million cases of COVID-19 have been reported in this age group during the pandemic [13]. This infection has the ability to make children extremely ill and require hospitalization, and infection complications can result in death in some cases [17]. However, the COVID-19 infection in children is milder than in adults [18,19,20,21].

On 21 December 2021, KSA started to vaccinate children aged between 5–11 years to protect them from getting the infection and reduce their morbidity and mortality resulting from COVID-19. This vaccination campaign also assisted them in resuming normal lives and attending school. In order to increase the rate of vaccination among children aged between 5 to 11 years old in KSA, vaccination hesitancy among their parents should be considered and assessed. Vaccination hesitancy is defined as unwillingness or reluctance to administer the vaccine or have one’s minors receive vaccine against infectious disease, although the vaccination has been approved to be effective and safe [22].

A vaccine that is both safe and effective is not enough to halt this pandemic; a high level of general public acceptance of vaccines is also required. Many studies have been conducted to evaluate the public perception of the COVID-19 vaccination and vaccination hesitancy [23]. Globally, there is a growing concern about vaccine hesitancy [24]. It has been found that factors associated with vaccination hesitancy among the general population around the globe are broadly classified as follows: (1) awareness and knowledge issues, (2) the risk–benefit ratio of vaccines, (3) gender, cultural, age, religious or socioeconomic reasons, and fear of the adverse effect of the vaccines [25,26,27,28,29,30,31]. Moreover, research has shown that vaccination hesitancy in adults was observed more in younger ages (in comparison with those aged 56 and more), females, individuals with low income, and that those who were not aware of the risk of infection were more likely to be hesitant to receive the vaccination [32,33,34]. Another study carried out in France demonstrated that vaccination hesitancy is multifactorial, and attributed it to concerns regarding the efficacy of the vaccine, various demographic factors, and the policy of the national vaccination program [35]. In KSA, a survey was conducted before the COVID-19 vaccine was licensed. The survey demonstrated that 67% of participants were willing to administer the hypothetical vaccination and 7% were hesitant to receive it [36]. An additional study was conducted in KSA on parental willingness to immunize minors aged between 12 to 18 years and it found that 44% of participants will immunize their children with a COVID-19 vaccination [37]. The most common (40.9%) factor for immunizing children was to protect other family members from contracting the COVID-19 infection. Concerns about the vaccine’s side effects were the most common reason for refusal (22.2%) [37].

Simultaneously with developing the COVID-19 vaccination plan for minors, it is significant to understand the reasons related to parental vaccination hesitancy and COVID-19 vaccination. Thus, the current study aims to determine the reasons associated with vaccination hesitancy among parents, and the prevalence and characteristics of the parents who are hesitant to allow their children aged between 5 and 11 years old to administer the COVID-19 vaccines.

## 2. Materials and Methods

### 2.1. Type of Study

This is cross-sectional study was carried out in KSA between May 2022 to September 2022, using a snowball sampling technique. A web-based questionnaire was created using Google Forms and distributed to study participants, i.e., parents, to define the proportion who reported vaccination hesitancy, their reasons behind it, in addition to determining their beliefs toward COVID-19 immunization. The questionnaire consisted of 15 questions and took approximately 7 min to answer all the questions. It included questions related to participants’ characteristics, participants’ experience and attitudes towards vaccination, factors affecting parents’ willingness to vaccinate their children, and reasons behind parental willingness or parental refusal to vaccinate their children. The online questionnaire was disseminated through social media Apps, for instance, Instagram, Twitter, Facebook, and WhatsApp. The invitation letter explained the objectives of our study as well as the estimated time needed to finish the survey. Responders will be kindly requested to the send questionnaire to their relatives and friends. Only 344 participants out of 400 responded completely to our questionnaire.

### 2.2. Selection Criteria

The eligibility criteria are parents who have minors aged between 5 to 11 years, and those who are residents of KSA during the time of questionnaire submission. We did not provide an age limit for responders. We targeted minors below 12 as this group is significantly affected by parental decisions in contrast to more mature children; furthermore, KSA recently they have included this age group in the COVID-19 campaign. If participants had more than one minor between the target age limit, they were requested to limit their responses to only one minor who was selected at random via the system.

We calculated the sample size using the Raosoft sample size calculator program and according to the total number of KSA population 35,761,605, based on a 95% confidence interval (CI); hence, the sample size was estimated to be 385 as the minimum.

### 2.3. Statistical Analysis

The data were analyzed by using GraphPad Prism 5 software (GraphPad Software, Inc., La Jolla, CA, USA). A *p*-value of <0.05 denoted statistical significance. The frequencies and descriptive statistics were used to express categorical data on the sociodemographic factors of the parents. To detect parental characteristics linked with their acceptance to immunize minors aged 5 to 11 years with COVID-19 vaccination, we used chi-squared analysis for categorical data.

### 2.4. Ethical Considerations

The study was conducted in accordance with the Declaration of Helsinki and approved by Taibah University College of Medicine Research Ethics Committee (IORG0008716—IRB00010413), May 2022. The online informed consent was taken from all responders who meet the eligibility criteria after completing screening questions. Responders clicked on the checkbox on the online screen to indicate their informed consent of participation.

## 3. Results

### 3.1. Participants’ Characteristics

Overall, 344 subjects participated in this study, of which 40.7% were male (n = 140), and 59.3% were female (n = 204). Most of the study participants were between 30 and 49 years old (67.4%), two-thirds were Saudi (n = 228), and almost half were educated to a bachelor degree level. The study participants were from across Saudi Arabia, with the highest percentage of 35.17% of the participants from Madinah (n = 121). Two-thirds of the study participants were employed either in the private sector (n = 114) or in the public sector (n = 124), with almost one-third of those employed being healthcare workers. The detailed characteristics of the study participants are described in Table 1.

### 3.2. Participants’ Experience and Attitudes towards Vaccination

The study participants were surveyed to examine their trusted information source and their previous vaccination experience. The majority of the participants (over 82%) indicated trust in information provided to them by healthcare (n = 284). On the other hand, information found in newspapers or magazines was least trusted (n = 50) by the study participants. One-third of the study participants (n = 115) previously received the seasonal influenza vaccine, while over three-quarters of them (n = 271) received the COVID-19 vaccination. Further information is found in Table 2.

### 3.3. Factors Affecting Participants’ Willingness to Vaccinate Their Children

Upon asking whether participants were willing to vaccinate their children with the COVID-19 vaccines or not, over one-third of them were willing to vaccinate their children (n = 130). Interestingly, almost half of the participants (n = 170) were resistant to the vaccination, and about 12.8% of them (n = 44) were unsure.

Several factors, personal and social, affected the participants’ willingness to vaccinate their children with the COVID-19 vaccines. The age of the parents was found to have a significant impact on their decision to vaccinate their children. Those between the age of 40–49 years of age were the most willing to vaccinate (almost 41%) compared to those 50 years or older who were most resistant to vaccination.

Other factors affecting the vaccination acceptance were participants’ gender, nationality, occupation, and being a healthcare worker. Female participants were more resistant to vaccinating their children (60%) compared to their male counterparts (40%). Saudis were more resistant to vaccinating their children (64.1%) compared to the non-Saudi participants (35.9%). Those private sector-employed parents were the most willing to vaccinate (43.8%), followed by those working in the governmental sector (35.3%). About 52.4% of non-healthcare workers were resistant to vaccinating their minors in comparison to healthcare workers (38.9%). A detailed description of the factors affecting participants’ willingness to vaccinate is described in Table 3.

### 3.4. The Impact of Previous Vaccination and Their Trust Source of Information on Participants’ Willingness to Vaccinate Their Children

Previous vaccination of the parents with either the seasonal flu (influenza) or COVID-19 vaccines positively affected their willingness to vaccinate. About 17.7% and 36% of those who were vaccinated with influenza and COVID-19 vaccines, respectively, were willing to vaccinate their children with the COVID-19 vaccines. On the other hand, 39.3% and 18.9% of those who were not vaccinated with the flu and the COVID-19 vaccines, respectively, were not willing to vaccinate their children with the COVID-19 vaccines.

The participants’ trusted source of information did not affect their willingness to vaccinate their children with the COVID-19 vaccines. Interestingly, almost half of those who trust healthcare workers as their sole source of information was not willing to vaccinate their children. The description of the participants’ attitude towards vaccination based on their previous vaccination and their trusted source of information is described in Table 4.

Table 5 and Table 6, list the reasons behind the parental willingness or refusal to vaccinate their minors. The most common reason that made the parents accept to vaccinate their minor was “I want to protect family members from contracting COVID-19” (35%), followed by “I am worried that my son/daughter will catch COVID-19. Moreover, the most common cause that made the parents of children aged 5 to 11 years to refuse their children was “I am concerned about the side effects of the vaccine” (37.4%), followed by “I do not think the vaccine prevents infection” (12.6%).

## 4. Discussion

The latest pandemic of COVID-19 took the world by storm, affecting every aspect of life globally. Saudi Arabia was not different from the rest of the world in being affected by it, despite the strict and progressive measures it took to limit the pandemic’s impact on the country [38]. Despite the local advances in identifying cost-effective prognostic markers of COVID-19 [39,40], COVID-19 vaccination was vital in tackling the pandemic and limiting its impact on Saudi public health. However, the public will to be vaccinated in Saudi Arabia was complex and differed across the country [41,42]. Upon the latest recommendation of COVID-19 vaccination for adolescents and children by the World Health Organization (WHO) [43], the issue of paternal willingness to vaccinate their children with the COVID-19 vaccines became central to public health policymakers.

The percentage of participants who have received the COVID-19 vaccine are similar to the officially reported vaccination coverage in Saudi Arabia [44]. Several studies attempted to identify the factors determining people’s hesitancy towards COVID-19 vaccination, both locally and internationally [25,26,27,28,29,30,31,45,46]. In our study, several factors were found to significantly impact parents’ willingness to vaccinate their children. The age was found to affect the willingness of participants to vaccinate their children, which was in line with the findings of Ennaceur and Al-Mohaithef and other studies [26,37,47]. Gender was another factor that significantly affected the COVID-19 vaccination willingness, in contrast to the previously described findings [37,41,48,49].

Interestingly, the level of parents’ education did not impact their willingness to vaccinate their children in our study. This finding was in line with what Sayed and Soares et al. described [41,50]. On the other hand, Bagateli described contradicting results, indicating a significant impact of parental education on their willingness to vaccinate their children [49]. Such conflicting results could be attributed to the demographic differences between the sampled populations, as the levels of education were similar between studies. Interestingly, no significant differences in parental willingness were observed between the different regions of Saudi Arabia, either in our study or others [34,37,41].

Expectedly, this study demonstrated that parents’ experience with vaccination—specifically, vaccination uptake—significantly affected their decision to vaccinate their children. Those who received either the flu (influenza) or COVID-19 vaccines were more likely to vaccinate their children. Almaghslah also described similar results in Saudi Arabia where participants varied in their vaccination status [51], which would subsequently affect their willingness to vaccinate. Furthermore, Aedh has described similar findings, in which parents’ willingness to vaccinate their children was significantly affected by their own previous COVID-19 vaccination [52].

Although every attempt was made to conduct and present the study in the best possible way, this study is not without limitations. The cross-sectional nature of the study limits the interpretation of the presented results to the sampled population at the time of the study’s conduction. Such a limitation may limit its comparability with other studies, which may have been conducted at different times in different circumstances. Additionally, the relatively small sample size of this study and the very small number of participants from certain regions could limit the generalizability of the study’s findings. A total of 344 participants from across Saudi Arabia participated in this study, whereas over 1400 participants participated in a similar study from a single region in Saudi Arabia [53].

## 5. Conclusions

The study presents several factors that affect the parental willingness to vaccinate their children in Saudi Arabia. These factors should be properly addressed when developing public health strategies to promote the COVID-19 vaccination of children in Saudi Arabia. Future studies should include a more participants from Saudi’s different regions to have a more representative results that could better direct public health policies in Saudi Arabia.

## Figures and Tables

**Table 1:** Characteristics of the study participants.

Characteristics	Number of Participants (%)
Total	344
Gender	Male: 140 (40.7%) Female: 204 (59.3%)
Age	20–29 years: 60 (17.5%) 30–39 years: 127 (36.9%) 40–49 years: 105 (30.5%) +50 years: 52 (15.1%)
Nationality	Saudi: 228 (66.3%) Non-Saudi: 116 (33.7%)
Educational Level	Up to High school Certificate: 60 (17.4%) Diploma *: 74 (21.5%) Bachelor’s degree: 166 (48.3%) Postgraduate: 44 (12.8%)
Region of Residence	Madinah: 121 (35.17%) Makkah: 59 (17.15%) Eastern Region: 31 (9.01%) Riyadh: 30 (8.72%) Tabuk: 23 (6.68%) Asir: 17 (4.94%) Al-Baha: 16 (4.65%) Najran: 14 (4.06%) Jazan: 10 (2.9%) Northern Borders: 9 (2.61%) Hail: 7 (2.03%) Qassim: 6 (1.74%)
Employment status	Unemployed: 82 (23.8%) Employed in the Private Sector: 114 (33.1%) Employed in the Public Sector: 124 (36%) Self-employed: 24 (7%)
Employment in the Healthcare sector	Yes: 77 (22.4%) No: 267 (77.6%)

* Diploma is an intermediate degree between high school and a bachelor’s degree, which could indicate vocational training post high school.

**Table 2:** The study participants’ trusted source of information and previous vaccination experience.

Experience	Number of Participants (%)
What is/are your trusted source(s) of information?	Healthcare workers: 284 (82.6%) Social Media: 124 (36%) ^ TV/Radio: 73 (21.2%) Friends/Family: 55 (16%) Newspapers/Magazines: 50 (14.5%)
Did you receive the seasonal influenza vaccine?	Yes: 115 (33.4%) No: 229 (66.6%)
Did you receive the COVID-19 vaccine?	Yes: 271 (78.8%) No: 73 (21.2%)

^ Percentage of the total participants.

**Table 3:** Participants’ characteristics and their impact on participants’ willingness to vaccinate their children.

Characteristics	Willing to Immunize the Child with COVID-19 Vaccine (n = 130, 37.8%)	Not Willing to Immunize the Child with the COVID-19 Vaccine (n = 170, 49.4%)	Not Sure (n = 44, 12.8%)	*p*-Value
Age				
20–29	22 (6.4%)	33 (9.6%)	4 (1.2%)	0.0018 **
30–39	48 (13.9%)	60 (17.4%)	19 (5.5%)	
40–49	43 (12.5%)	42 (9.7%)	20 (5.8%)	
50 and more	17 (4.9%)	34 (9.9%)	1 (0.3%)	
Gender				
Female	66 (19.2%)	102 (29.6%)	36 (10.5%)	0.0014 **
Male	64 (18.6%)	68 (19.8%)	8 (2.3%)	
Nationality				
Saudi	83 (24.1%)	109 (31.7%)	36 (10.5%)	<0.0001 ***
Non-Saudi	47 (13.7%)	61 (17.7%)	8 (2.3%)	
Education level				
Secondary school or less	19 (5.5%)	31 (9%)	10 (2.9%)	0.1012 (ns)
Diploma	27 (7.8%)	42 (12.2%)	5 (1.4%)	
Bachelor’s Degree	73 (21.2%)	72 (20.9%)	21 (6.1%)	
Higher Degree	11 (3.2%)	25 (7.3%)	8 (2.3%)	
City of residence				
Western	63 (18.3%)	81 (23.5%)	29 (8.4%)	0.3411 (ns)
Eastern	13 (3.8%)	13 (3.8%)	5 (1.4%)	
Southern	25 (7.3%)	38 (11%)	3 (0.9%)	
Northern	13 (3.8%)	18 (5.2%)	2 (0.6%)	
Central	16 (4.6%)	20 (5.8%)	5 (1.4%)	
Occupation				
Governmental sector	46 (13.8%)	58 (16.9%)	20 (5.8%)	0.0004 ***
Private sector	57 (16.6%)	50 (14.5%)	7 (2%)	
Self-occupation	9 (2.6%)	15 (4.4%)	0 (0%)	
Not working	18 (5.2%)	47 (13.7%)	17 (4.9%)	
Working in the healthcare system				
Yes	42 (12.2%)	30 (8.7%)	5 (1.4%)	0.0018 **
No	88 (25.6%)	140 (40.7%)	39 (11.3%)	

The percentages included in the table indicate the percentage of the total number of participants. ** Denotes a *p*-value of <0.01 *** Denotes a *p*-value of <0.001. ns: not significant.

**Table 4:** Participants’ willingness to vaccinate based on their previous vaccination and trust source of information.

	Willing to Immunize the Child with the COVID-19 Vaccine	Not Willing to Immunize the Child with the COVID-19 Vaccine	Not Sure	*p*-Value
Administered Flu vaccine	61 (17.7%)	35 (10.2%)	19 (5.5%)	<0.0001 ***
Not administered Flu vaccine	69 (20%)	135 (39.3%)	25 (7.3%)	
Administered COVID-19 vaccine	124 (36%)	105 (30.5%)	42 (12.2%)	<0.0001 ***
Not administered COVID-19 vaccine	6 (1.7%)	65 (18.9%)	2 (0.6%)	
Trusted source of information				ns
Health care workers	47 (13.7%)	64 (18.6%)	20 (5.8%)	
Social media	8 (2.3%)	19 (5.5%)	2 (0.6%)	
T.V/Radio	3 (0.9%)	4 (1.2%)	3 (0.9%)	
Family and friends	2 (0.6%)	5 (1.4%)	1 (0.3%)	
Journals and newspapers	1 (0.3%)	3 (0.9%)	0 (0%)	
multiple	71 (20.6%)	73 (21.2%)	18 (5.2%)	

The percentages included in the table indicate the percentage of the total number of participants. *** Denotes a *p*-value of <0.001. ns: not significant.

**Table 5:** Reasons behind parents willing to vaccinate their children n= 174.

I want to protect family members from contracting COVID-19	61 (35%)
I am worried that my son/daughter will catch COVID-19	37 (21.3%)
The physician advised me to vaccinate my son/daughter with the COVID-19 vaccine	30 (17.2%)
My son/daughter has a chronic disease (such as asthma, diabetes, etc.) that requires a COVID-19 vaccine.	31 (17.8%)
There are many cases of COVID-19 in my social surroundings	15 (8.6)

**Table 6:** Reasons behind the parental refusal to vaccinate their children n = 214.

I am concerned about the side effects of the vaccine	80 (37.4%)
I do not think the vaccine prevents infection	27 (12.6%)
The efficacy of vaccines is unknown	30 (14%)
I do not think COVID-19 causes serious illness in children	22 (10.3%)
The vaccine is very new	10 (4.6%)
My child is immune to COVID-19 because he has had it before	7 (3.3%)
I find it annoying to take more than one dose of the COVID-19 vaccine	18 (8.4%)
I avoid most vaccinations	8 (3.7%)
My child does not suffer from any chronic diseases	6 (2.3%)
I do not have medical insurance	5 (2.3)
I do not think my child will get COVID-19	1 (0.5)

## Data Availability

The survey data that support the findings of this study are available from the corresponding author, upon reasonable request.
